# *Lactococcus lactis* as a safe and inexpensive source of bioactive silver composites

**DOI:** 10.1007/s00253-017-8443-x

**Published:** 2017-08-25

**Authors:** Railean-Plugaru Viorica, Pomastowski Pawel, Meller Kinga, Złoch Michal, Rafinska Katarzyna, Buszewski Boguslaw

**Affiliations:** 1 Department of Environmental Chemistry and Bioanalytics, Faculty of Chemistry, Nicolaus Copernicus University, Gagarina 7, 87-100 Torun, Poland; 2 Interdisciplinary Centre of Modern Technology, Nicolaus Copernicus University, Wileńska 4, 87-100 Torun, Poland

**Keywords:** Silver composites, *Lactococcus lactis*, Antibacterial activity, Cytotoxicity, Biosynthesis

## Abstract

This research develops a safe, inexpensive, and more accessible source for synthesis of silver nanoparticles. The bioactive silver composites synthesized by *Lactococcus lactis* 56 KY484989 (LCLB56-AgCs) were characterized by various physico-chemical techniques and investigated for their antimicrobial activity and cytotoxicity. The average amount of nanoparticles was 0.363 ± 0.09 mg from 50 mL of culture medium. The synthesis efficiency varied from 71 to 85%. Synthesized silver nanoparticles with spherical in shape were found to be of 5–50 nm and average diameter 19 ± 2 nm. Based on the shape of isotopic pattern of d-electrons metals, the signals of silver isotopes [^107^Ag]^+^ at *m*/*z* 106.905 and [^109^Ag]^+^ at *m*/*z* 108.910 were confirmed. Moreover, LCLB56-AgCs exerted an inhibitory effect against all tested bacterial strains (*Pseudomonas aeruginosa* ATCC10145, *Proteus mirabilis* ATCC25933, *Staphylococcus epidermidis* ATCC49461, *MSSA* ATCC29213, and *Staphylococcus aureus* ATCC6338). More pronounced antimicrobial effect was noticed for 15 μg/well. Minimum inhibitory concentration required to inhibite the growth of 90% organism (MIC_90_) of synthetized LCLB56-AgCs was in a range of 3.125–12.5 μg/mL. The concentration at which the viability of the L929 cells was reduced to 50% was above 200 μg/mL for LCLB56-AgNCs. These results open up possibilities for many applications of bioactive silver composites (BioAgCs) synthesized by *L. lactis* 56 in food and pharmaceutical industries.

## Introduction

Nanotechnology involves the study of structures of size to ca. 100 nm, which possess new properties and functions. Some of these new nanomaterials, especially those containing silver, exhibit a very efficient antibacterial effect. Currently, a lot of research refers to silver in the form of nanoparticles. They possess many advantages, i.e., exhibit antibacterial effect against a broad range of microorganisms (bacteria, fungus, parasites) and usually in antibacterial doses do not exert cytotoxic effect toward eukaryotic cells. These properties are very important in association with the emergence of pathogenic bacterial strains that possess a resistance even toward several antibiotics (Cherusova and Epple 2013; Singh et al. 2015a, b). Hence, the development of new antimicrobial solutions has become of increasing importance for medicine. Silver nanoparticles/composites in different forms constitute a very promising approach for the development of new antibacterial therapies against of the skin, throat, or ear infections.

There are physical and chemical methods available for synthesis of silver nanoparticles. Moreover, during the recent years, a wide range of metal composites, doped silver, and polymer/silver nanocomposites are a subject of increased interest (Humberto 2014, Ciobanu et al. 2015). However, most of them demand hazardous chemicals, toxic byproducts, and high energy consumption (Cherusova and Epple 2013). Recently, biological methods of silver nanoparticles synthesis using green algae, bacteria, fungi, and plant extracts are gaining impetus due to their low costs and simplicity (Singh et al. 2013; Salunke et al. 2014). The properties of synthesized nanoparticles depend on used bacterial strain, type of medium, pH, and temperature. In this work, for the synthesis of bioactive silver composites, we chose *Lactococcus lactis* which belongs to both lactic acid bacteria (LAB) and probiotic groups.

Lactic acid bacteria are a clad of Gram-positive non-sporing cocci or rods with non-aerobic habit which produce lactic acid as the major metabolic end product of carbohydrate fermentation (Nejati et al. 2016). These microorganisms are found in milk and fermented products and in fermented vegetables and beverages. They inhibit the growth of pathogenic and deteriorating microorganisms. Most of them are residents of gastrointestinal microbiota which provides a microbial barrier against microbial pathogens. They can elicit innate and adaptive immune response by binding to specific receptors on immune cells (Quinto et al. 2014). Moreover, lactic acid bacteria produce bacteriocins that are small ribosomally synthesized proteins which possess antibacterial activity. They may act via pore forming, nuclease activity, or peptidoglycan production inhibition. Many studies proved that the application of bacteriocins is efficient barrier against pathogens.

The aim of this study was to develop inexpensive, simple, and fast method to synthesis of silver composites for future use in the food and pharmaceutical industries. Previous research indicated that biomass of lactic acid bacteria is a good reducing and capping agent for the efficient production of silver nanoparticles (Sintubin et al. 2009; Matei et al. 2015). In our study, we used supernatant of *L. lactis* liquid culture. The advantage of proposed method is the safe application of known to man LAB strain. Moreover, this strain is easy and not expensive to culture. The obtained LCLB56 composites were characterized by various physico-chemical techniques and investigated for their antimicrobial activity and cytotoxicity.

## Material and methods

Effectiveness of LCLB56-AgCs was studied against following bacteria: *Pseudomonas aeruginosa* ATCC10145, *Proteus mirabilis* ATCC25933 (Collection of the Collegium Medicum of Nicolaus Copernicus University), *S. epidermidis* ATCC49461 from the collection of Centre for Modern Interdisciplinary Technologies, Nicolaus Copernicus University, Torun, *MSSA* ATCC29213 (methicillin-sensitive *Staphylococcus aureus*), and *S. aureus* ATCC6338 from Sanitary-Epidemiological Station in Torun*.* Mueller–Hinton (MH) broth was purchased from Sigma-Aldrich (Germany), and a solution of phosphate buffered saline (PBS-10X) was supplied from GenoPlast (Poland). L929 mouse Cell Line from European Collection of Authenticated Cell Cultures. Dulbecco’s modified Eagle medium (DMEM), glutamine, fetal bovine serum (FBS), and dimethyl sulfoxide (DMSO) were from Sigma-Aldrich. MTP Anchor Chip 384 target (Bruker Daltonik, Bremen, Germany) was used in matrix-assisted laser desorption ionization–time of flight (MALDI–TOF MS) experiments, as well as chemicals from Sigma-Aldrich. The milk was supplied by Dairy Factory in Drzycim, Poland. Water was purified using a Milli-Q RG system by Millipore (Millipore Intertech, Bedford, MA, USA).

### Isolation of bacteria from milk products

The samples of milk were plated on M17 medium and incubated at 37 °C for 24 h. Then, the same combination of media was streaked with the obtained biological material using sterile inoculation loop and incubated at 37 °C for 24 h. Subsequently, the grown colonies were applied for the preparation of dilutions in the range of 10^−1^ to 10^−8^ using sterilized 0.87% KCl and double distilled water (H_2_Odd). All dilutions were plated (1 mL of inoculum) onto Petri dishes with culture media (M17) and then incubated at 37 °C for 24 h. Based on the visible morphological characteristics (i.e., color and texture of colony as well as shape, size and Gram staining of cells), one isolate was chosen for further analysis.

### Identification of the isolated bacterial strain by 16S rDNA PCR

For molecular identification, bacterial DNA was extracted from overnight cultures (TSB medium, 37 °C) using the kit for the isolation of genomic DNA from bacteria EXTRACTME (DNA Gdańsk, BLIRT S.A, Poland). DNA concentrations were determined using a UV–Vis spectrophotometer (NanoDrop 2000). Fragments of 16S ribosomal DNA (rDNA) were amplified using the primers 27F (5-AGAGTTTGATCMTGGCTCAG-3) and 1492R (5-GGTTACCTTGTTACGACTT-3). An amplification reaction was performed in a thermocycler ABI 9700 (Applied Biosystems™, USA) with the use of thermostable polymerase OptiTaq (EURx, Poland). Subsequently, PCR products were purified using the ExoSAP-IT PCR Product Cleanup Kit (Affymetrix, Inc., USA). Direct sequencing of PCR products was performed using 341F (5-CCTACGGGAGGCAGCAG-3), 518R (5-GTATTACCGCGGCTGCTGG-3), and 928F (5-TAAAACTYAAAKGAATTGACGGG-3) primers, BigDye Terminator Mix v3.1 and genetic analyzer ABI3730xl (Applied Biosystems™, USA). From obtained sequences, it was assembled the contig and then consensus sequences were compared with known 16S rDNA genes at the National Center for Biotechnology Information (NCBI) BLAST database (Altschul et al. 1997). Obtained sequence was submitted to GenBank, and it was received the accession number.

### Identification of the isolated bacteria by intact cell MALDI–TOF MS

The matrices HCCA (10 mg/mL) was prepared in bacterial solution (EtOH/ACN/H2O, 1:1:1 (*v*/*v*/*v*)). The trifluoroacetic acid (TFA) solution was added to Bacterial Solution with 2.5% *v*/*v* of the final concentrations. Then, under sterile conditions, two lapfuls of bacterial cells were suspended in 5 μL of bacterial solution and thoroughly vortexed for 30 s. Two microliters of bacterial suspension was mixed with 2 μL of the matrix, and then 1 μL of the mixture was overlaid on the ground steel MALDI target. After 30 min, when all spots had dried, the target was placed in the ultrafleXtreme MALDI–TOF/TOF mass spectrometer for measurement according to Pomastowski et al. (2015). The ultrafleXtreme MALDI–TOF/TOF mass spectrometer is equipped with a modified neodymium-doped yttrium aluminum garnet (Nd:YAG) laser (Smartbeam IITM) operating at the wavelength of 355 nm and the frequency of 2 kHz. IC MALDI–TOF MS spectra were recorded manually in linear positive mode within *m*/*z* range of 300–30,000 and applying the acceleration voltage of 25 kV. All the mass spectra were acquired and processed with the dedicated software: flexControl and flexAnalysis, respectively (both from Bruker).

### Synthesis of LCLB56-AgCs

For synthesis of the silver composites, 250 mL MH broth was prepared in a flask, sterilized and inoculated with a fresh culture of LCLB56 strain. The inoculate was transferred to bioreactor Biostat A (Sartorius Stedim Biotech, Germany) and incubated (100 rpm) with MH medium at 26 °C for 5 days. Then, the culture was centrifuged at 9000 rpm for 15 min, the supernatant was combined with 1 mM AgNO_3_ (final concentration) and incubated at 26 °C in a shaker for 7 days in the dark. Bioactive composites were centrifuged at 14,000 rpm for 30 min to concentrate. Then, unreacted silver ions and low molecular weight metabolites were removed by a 3-day dialysis (3 kDa cutoff, Spectrum Lab, USA). MH broth mixed with 1 mM AgNO_3_ (final concentration) was used as a control sample.

### Characteristics of synthesized LCLB56 biosilver composites

#### DLS and ZP

The size and zeta potential measurements of LCLB56-AgCs were performed using Zetasizer Nano Series (Malvern Instruments, Great Britain). Size distribution analysis was measured by the dynamic light scattering methods. First, the BioAgCs was suspended in a 0.87% KCl solution at pH 5–7; then, the solution was sonicated for 20 min and vortexed directly before measurements. It was used UV-grade cuvettes for size measurements and folded capillary cells for zeta potential determination. All measurements were analyzed in triplicate.

#### FTIR analysis

FTIR analysis of surfaced functional groups in organics part of silver biocolloids has been performed. The samples were dried at 37 °C to remove the water. The infrared spectrum was registered three times in MIR range (FTIR Genesis II Mattson, USA) using the pellet method in KBr. Spectroscopic data were processed using WINFIRST software. The sample of obtained silver composites was also suspended in 0.09% NaCl at pH 4.7 and 10. The FTIR spectra were registered in range of 1350–1870/cm using thin layer method in DirectDetect® Infrared Spectrometer (Merck Millipore, Germany).

#### Electron microscopy and X-ray diffraction study

The size of synthesized LCLB56-AgCs size was measured using transmission electron microscopy (TEM, FEI Tecnai F20 X-Twin) and scanning electron microscopy (SEM, LEO 1430VP) in tandem with EDX detector (XFlash 4010, Bruker AXS). A sample solution was applied to a carbon-coated copper grid. Then, the sample was subjected to drying. X-ray analysis diffraction (XRD) was used for determination and characterization of the crystal structure. The LCLB56-AgCs sample was deposited onto the glass slide and then recorded by X-ray diffractometer (X’Pert Pro Analytical Phillips) equipped with Ni filter and Cu Kα (*λ* = 1.54056 Å) radiation source.

#### Fluorescence spectroscopy

Fluorescence spectra were obtained with a spectrofluorometer Jasco FP-XX (Japan). Samples 0.12 mg and 1.2 mg of silver nanocomposites were suspended in 1 mL of deionized water, sonicated for 5 min, and positioned in a quartz cuvette with a 1-cm path length. Three-dimensional (3D) excitation-emission spectra were recorded with 1 nm wavelength intervals in range 250–585 nm and 260–600 nm, respectively.

#### MALDI–TOF/TOF MS analysis

All chemicals for the MALDI MS analyses were supplied at the highest commercially available purity by Fluka Feinchemikalien (Neu-Ulm, Germany; a subsidiary of Sigma-Aldrich). Ground steel targets (Bruker Daltonik, Bremen, Germany) were used for sample deposition. The α-cyano-4-hydroxycinnamic acid (HCCA) was employed as matrix for MALDI analysis of biosilver spotted by dried droplet method (Pomastowski et al. 2015). Protein Calibration Standards I (Bruker Daltoniks, Bremen) and HCCA were used for external calibration. All the MS spectra were obtained using the MALDI–TOF/TOF mass spectrometer (Bruker Daltonik, Bremen, Germany) equipped with a modified Nd:YAG laser operating at the wavelength of 355 nm and frequency of 2 kHz. The system was controlled using the Bruker Daltonik software (flexControl and flexAnalysis). Molecular fingerprint (MF) spectra of silver biocolloids were recorded in reflectron positive mode, within an *m*/*z* range of 100–3500, and applying an acceleration voltage of 25 kV.

### Antimicrobial activity

#### MIC method

The assay was performed according to Clinical and Laboratory Standards Institute (CLSI) guidelines. The final concentration of LCLB56-AgCs (200, 100, 50, 25, 12.5, 6.25 and 3.125 μg/mL) was added to bacterial strains previously cultured on Mueller–Hinton broth (1 × 10^6^ CFU/mL). The plates were incubated for 18 h at 35 °C. Bacterial cells viability was read at 600 nm using BIOLOG multimode reader. Untreated bacterial materials served as control.

#### Well-diffusion method

Well diffusion method has been performed on Mueller–Hinton agar (MHA) plates. The silver composites (15, 7.5, and 1.875 μg/well) were aseptically placed on the MHA surface inoculated with 100 μL of 1 × 10^6^ bacterial suspension. Plates were incubated at 35 °C for 24 h, and then the diameter of the inhibition zones was measured in mm. All measurements were carried out in triplicates.

#### Fluorescence microscopy assay

The antimicrobial effect of LCLB56-AgCs was also observed microscopically against *S. aureus* using fluorescence dyes (acridine orange (0.12 μg/mL) and ethidium bromide (0.4 μg/mL) in order to visualize living and dead cells. Viability of cells exposure to different doses (12.5 and 100 μg/mL) of silver composites was detected through the filter set to 43 He and 38 using Zeiss Axiocom D1 fluorescence microscope. LCLB56-AgCs was added to cell culture media (1 × 10^6^ of cells) and then analyzed after 3 and 24 h of incubation. Stained treated cells were incubated at room temperature for 5 min in the dark, centrifuged at 4000 rpm for 5 min, and then the supernatant was discarded to eliminate the unbound dyes. The remaining cell pellet was resuspended in sterile phosphate-buffered saline. The cells untreated with silver composites served as control.

#### Cytotoxicity assay

To determine cell viability after treatment with silver biocomposites, the colorimetric MTT metabolic activity was used. L929 cells (1 × 10^4^ cells/well) were chosen to study cytotoxic potential as ISO 10993–5 recommends. L-929 fibroblast cells were grown in DMEM with 2 mM glutamine and 10% fetal bovine serum (FBS) in a 96-well plate at 37 °C and exposed to varying concentrations of LCLB56-AgCs (12.5–200 μg/mL) for 24 and 48 h, respectively. The cells untreated with silver composites served as negative control. Then MTT solution was added to the final concentration 0.2 mg/mL. After 3.5 h of incubation at 37 °C, media were carefully removed and DMSO was added. Samples were agitated on orbital shaker for 15 min. The absorbance was read at 590 nm by a microplate reader. All experiments were performed in triplicate, and the relative cell viability (%) was expressed as a percentage of untreated control cells. Concentration of LCLB56-AgCs showing a 50% reduction in cell viability (i.e., IC50 values) was calculated.

##### Nucleotide sequence accession number

The nucleotide sequences of 16S rDNA *L. lactis* 56 has been submitted in GenBank with accession numbers KY484989 and deposited in the Polish Collection of Microorganisms (PCM) under deposit no. B/00116.

## Results

### Identification of the LAB strain isolated from milk

**Fig. 1:**
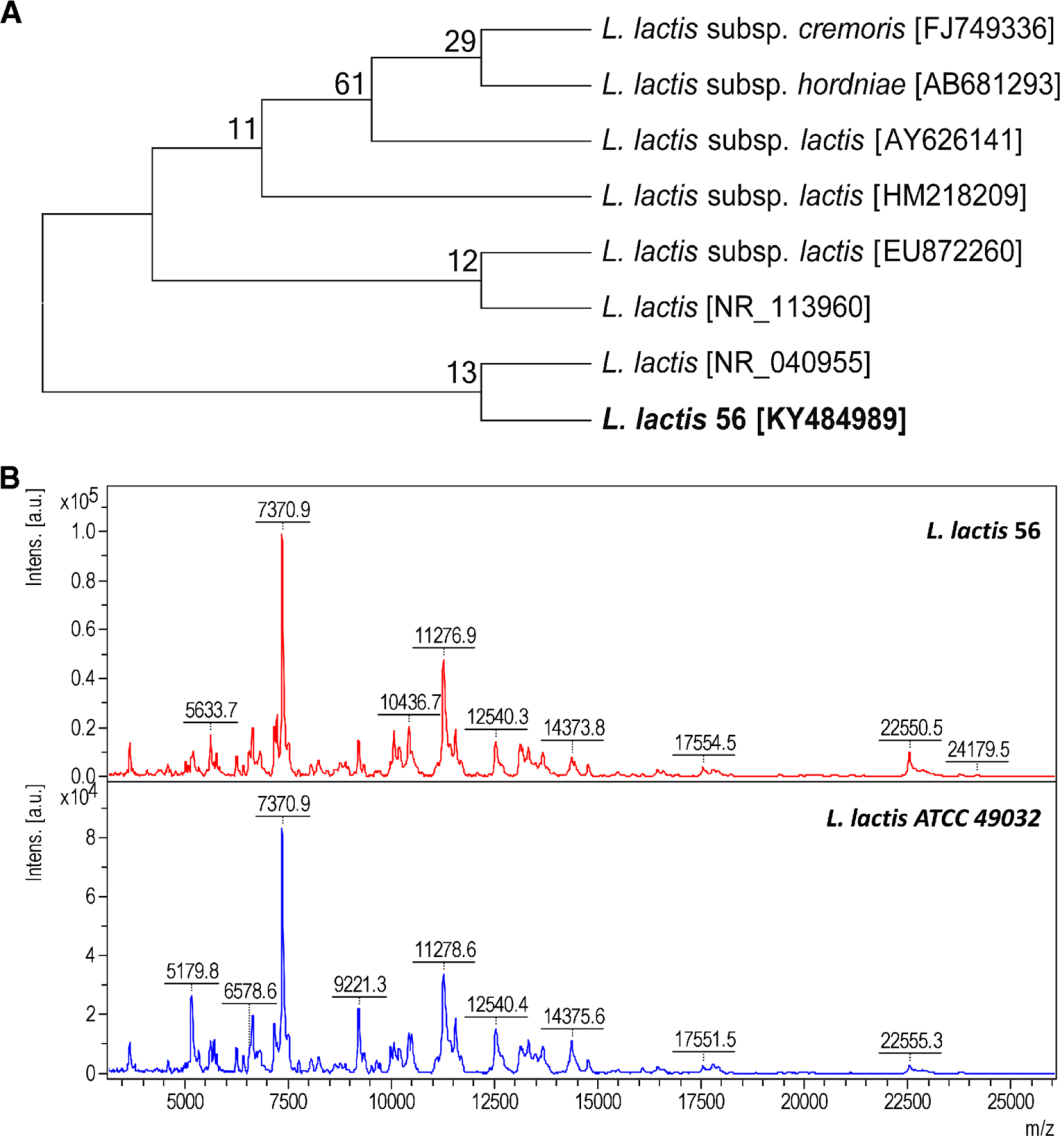
**a** Phylogenetic relationships of *Lactococcus lactis* 56 strain. Neighbor-joining analysis of 16S rDNA sequences, using the number of differences method (Nei and Kumar 2000) combined with bootstrap analysis from 1000 replicates (bootstrap values < 50% not shown). All positions containing gaps and missing data were eliminated. There were a total of 1440 positions in the final dataset. Analyses were performed using MEGA7 software (Kumar et al. 2016). **b** The confirmation spectrum of *L. lactis* 56 performed using the MALDI–TOF MS technique on HCCA matrix

The obtained results after 16S rDNA sequencing of LAB strain investigated in this study was identified as *L. lactis* 56 [KY484989] with 100% (1469/1469) sequence overlap with the most similar *L. lactis* NCDO 604 [NR_040955] (Fig. 1a). Moreover, the acquired spectra for *L. lactis* 56 were compared with MALDI spectra of reference bacterial strains (*L. lactis* ATCC 49032). 99–100% of cover was obtained Fig. 1b.

### Electron microscopy and X-ray diffraction study

**Fig. 2:**
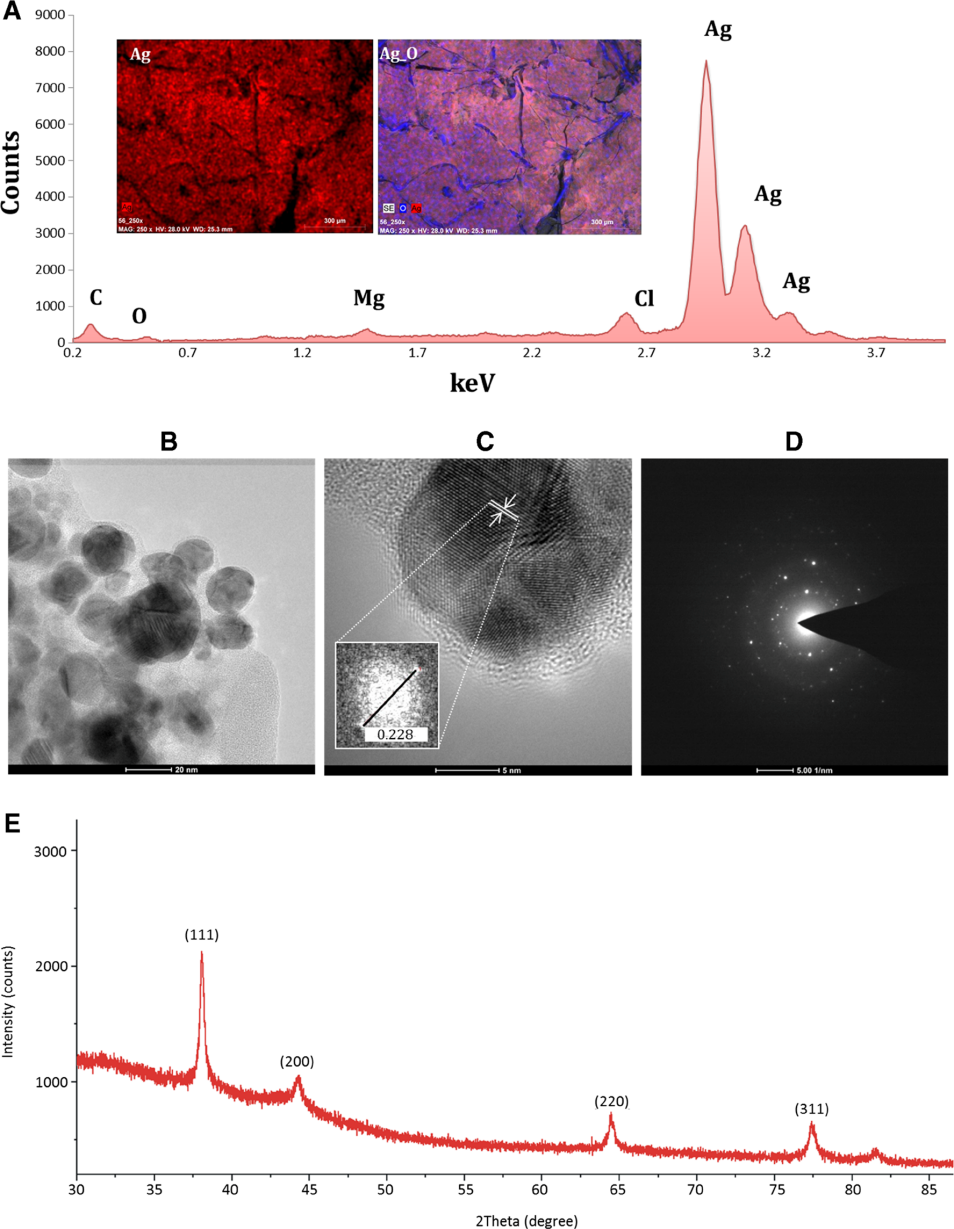
EDX spectra of a LCLB56-AgNCs (**a**), TEM micrograph (**b**), and FFT image (**c**); SAED (**d**) and XRD (**e**) patterns of bioactive silver nanoparticles

EDX spectrum shows a signals in range of 2.7–3.2 keV confirms the presence of metal silver and binding energies of biocompounds on the surface of the Ag-NCs. (Fig. 2a). The other small peaks correspond to elemental carbon, oxygen, sodium, magnesium and chloride related with the presence of organics part and remains of the medium onto silver nanoparticle surface. TEM analysis revealed the presence of many spherical in shape nanoparticles with size of 5–50 nm. Their average diameter was 19 ± 2 nm. A high-resolution TEM image showed interference fringe patterns with interplanar distances 0.228 nm characteristic for silver (Fig. 2b, c). The selected area diffraction pattern (SAED) confirmed highly crystalline nature of synthesized nanoparticles. Figure 2d shows concentric rings which can be assigned to the diffraction planes (111), (200), (220), and (311) characteristic for metallic silver.

Figure 2e shows X-ray analysis diffraction (XRD) pattern of silver composites deposited on a glass slide. Results confirm that the silver particles formed in our experiments are in form of nanocrystals. Typical peaks were present at 2*θ* = 38.08°, 44.34°, 64.43°, and 77.41° which corresponds to (111), (200), (220), and (311) Bragg reflections characteristic for silver. Full width at half maximum (*β*) of the first peak measured in XPert High Score program was 0.3103. Hence, the size of silver nanoparticles calculated according to Debye–Scherrer equation was approximately 27.18 nm. The Debye–Scherrer used formula was

1 $$ \boldsymbol{D}=\frac{\boldsymbol{K}\boldsymbol{\lambda }}{\boldsymbol{\beta}\ \mathbf{\cos}\ \boldsymbol{\theta}} $$

***D***—crystallite size; ***λ***—the wavelength of the X-ray source (0.15418 nm); ***β***—the full width at half maximum of the diffraction peak; ***K***—Scherrer constant with a value from 0.9 to 1; ***θ***—Bragg’s diffraction angle.

### FTIR

**Fig. 3:**
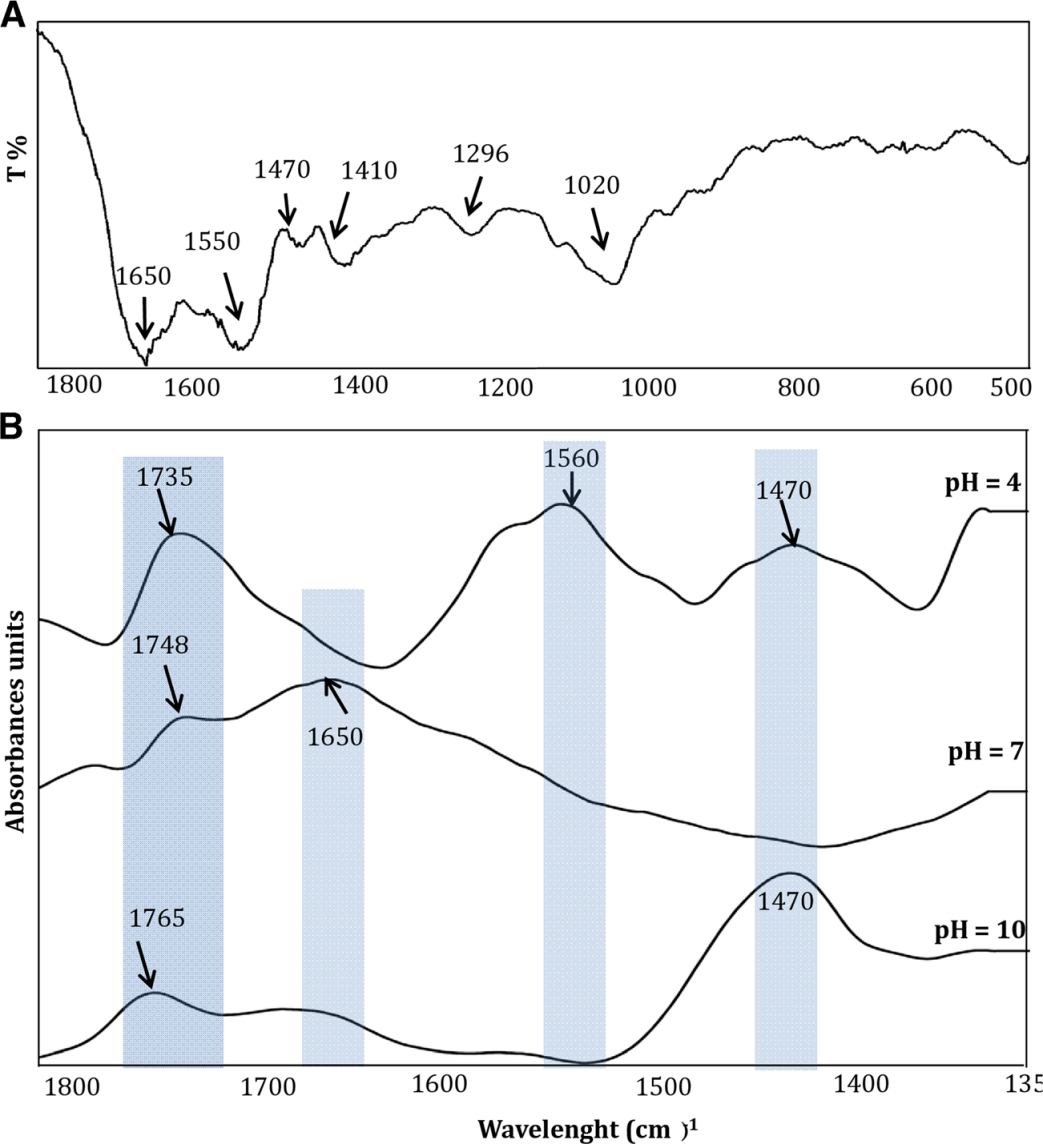
Infrared spectrum of LCLB56-AgNCs registered in MIR range (**a**) and using thin layer method in DirectDetect® Infrared Spectrometer (**b**)

Spectroscopic study in infrared range 1800–500/cm was illustrated to identified functional groups of compounds localized in organics deposit of the silver biocolloids. Figure 3a illustrates FTIR spectra of dried biosilver composites in KBr disc. The absorption bands at 1650/cm are a consequence of the presence of amide I vibrations arisen mainly from the C=O stretching vibration, whereas amide II region (N–H bending and C–N stretching) occurred at 1550/cm (Haris and Severcan 1999). The spectral bands at 1650/cm (Fig. 3b) can indicate at symmetric stretching vibration from arginine residues (CN_3_H_5_^+^) (Sreeprasad et al. 2011). This amino acid constitutes the active carboxyl groups, and the FTIR spectra (Fig. 3b) suggest that it is involved in the ionization processes of organics part at pH range 4–10 (Haris and Severcan 1999; Sreeprasad et al. 2011). Moreover, change in spectrum band at range 1560/cm (Fig. 3b) indicates the presence of glutamic and aspartic acid (–COO^−^) in colloids structure. The bands at 1560/cm (Fig. 3a, b) originate from deprotonated carboxyl groups and indicate participation of acid amino acid such as glutamic and aspartic acids in the structure of organics deposit of silver biocolloids (Sreeprasad et al. 2011). The absorption peaks localized at 1560 (Fig. 3a, b) are also related to amide II vibrations (Sreeprasad et al. 2011). The spectral band at 1470/cm (Fig. 3a, b) could originate from in plane bending of methylene (–CH_2_) group. Finally, this band is characteristic for His^−^ as well as bending from methyl (CH_3_) group and stretching from CN group (Sreeprasad et al. 2011). The p*K*a value of imidazole group is 6. Therefore, the change at spectra range could indicate the presence of phenolic ring and deprotonation processed depends on pH of solvents (Fig. 3b). The band at 1410 and 1020/cm (Fig. 3a) corresponds to bending vibration of hydroxyl groups ν (OH) and C–O–C vibration band of lactic acid, respectively. The absorption band at 1296/cm (Fig. 3a) originates from carbonyl (–C=O) banding vibration of lactic acid and its metabolites (Vodnar et al. 2010). The shift of absorption band at 1735, 1748, and 1765/cm indicates on present C=O stretching vibrations of fatty acids or lipids (Sreeprasad et al. 2011). The change in spectra (Fig. 3b) results from pH-dependent reaction of carboxyl groups. The band in range of 800–600/cm (Fig. 3a) is characterized by C–H vibrations of proteins, peptides, amino acids, or lipids in organic deposit (Sreeprasad et al. 2011).

### Fluorescence spectroscopy

**Fig. 4:**
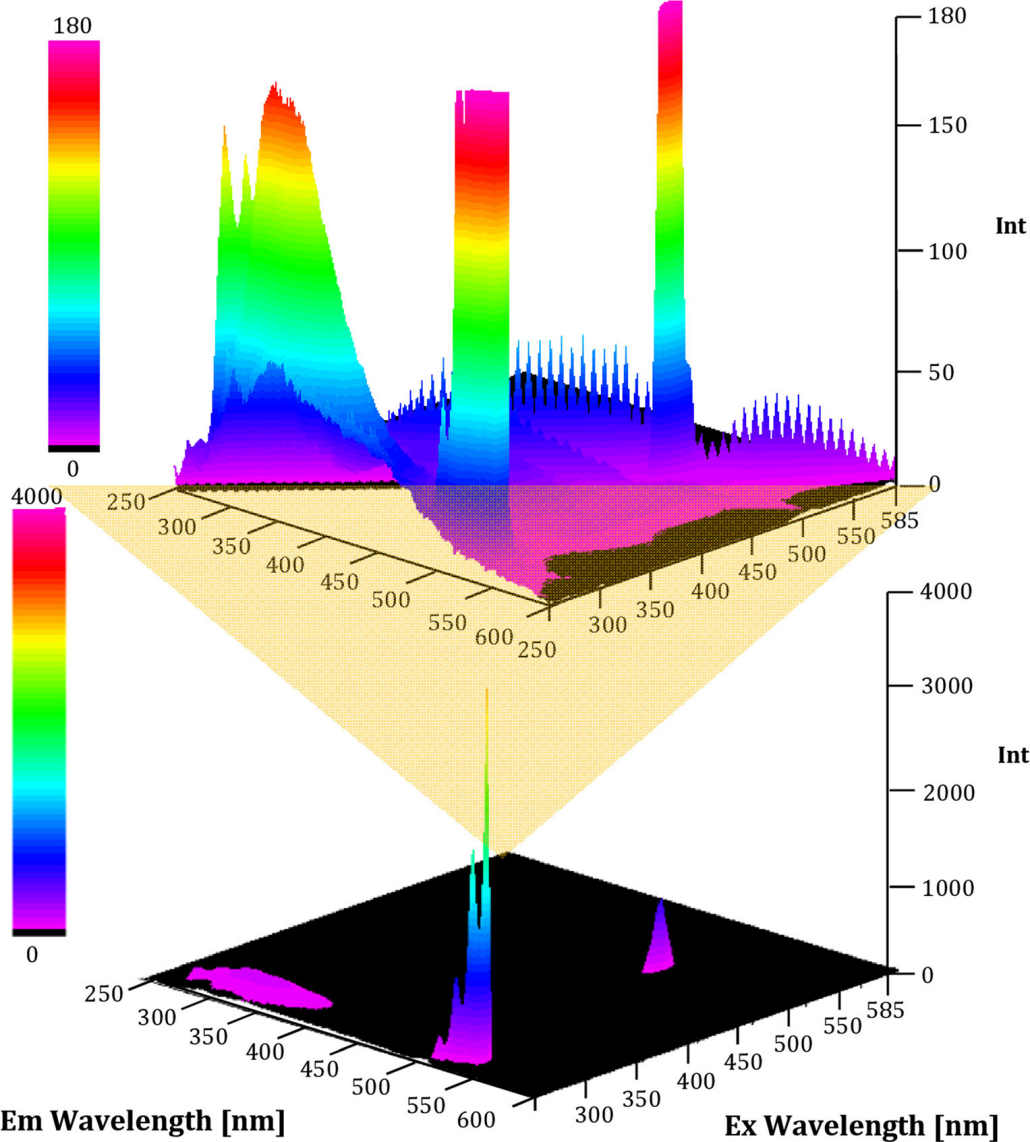
Fluorescence of LCLB56-AgNCs nanocomposites

The 3D profiles (Fig. 4) of silver nanocomposites were recorded to determine the fluorescence nature of synthesized biocolloids. There were observed three main groups of florescence bands. The most intensive fluorescence band was measured at excitation and emission wavelengths of 270 and 540 nm, respectively. It is caused by florescence of quartz consist of the cuvette, used for fluorescence assay. In contrast to negative control (water) it was observed the increase of florescence intensity in case of suspension of silver nanocomposites. Moreover, the registered bands at excitation and emission wavelengths were of 480 and 493 nm and 280 and 320 nm, respectively (Fig. 4). Furthermore, in was measured the fluorescence of silver nanocomposites in two different concentrations. In case of silver nanocomposites at concentration 0.12 mg/mL, the fluorescence intensity of all three bands was at the similar level in comparison to silver nanocomposites at concentration 1.2 mg/mL.

### Matrix-assisted laser desorption ionization–time of flight mass spectrometry

**Fig. 5:**
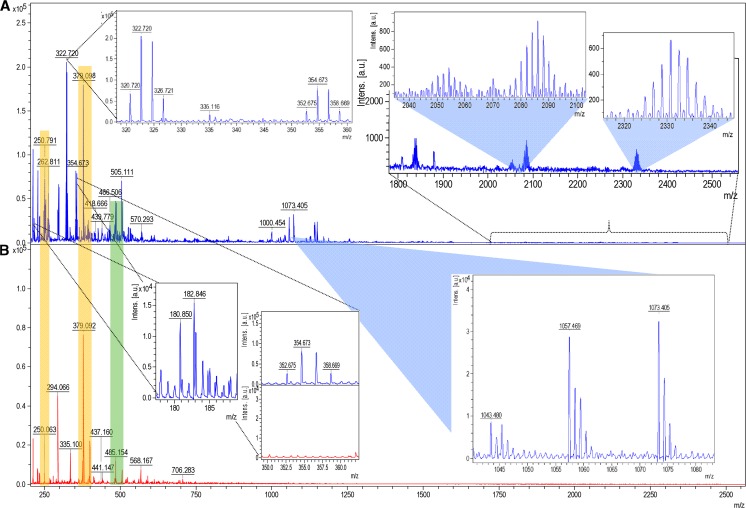
Molecular fingerprint of synthesized LCLB56-Ag biocolloids (**a**) using MALDI–TOF MS technique and HCCA matrix with spotted 1 mM silver nitrate as control (**b**)

Spectrometric matrix-assisted laser desorption ionization–time of flight (MALDI–TOF MS) analysis was performed to illustrate the presence of silver isotopes, silver-organic connections and clusters of silver in analyzed sample of silver biocomposites. Figure 5a illustrates molecular fingerprint of synthesized silver biocolloids, registered in refection and positive mode. To avoid the false positive results, HCCA matrix with spotted 1 mM silver nitrate was used as a control (V) (Fig. 5b). Many of matrix signals are present in LCLB56, e.g., *m*/*z* 190.050 origin from ionized HCCA matrix [HCCA + H]^+^, but *m*/*z* 379.092 (yellow stripe) results from ionized dimer of matrix [2HCCA + H]^+^ (Hendrik et al. 2004). Signals *m*/*z* 527.097, 1043.480, 1057.469, 1073.405, and 1150.130 are characteristic for ionized peptides [P–H]^+^. Peptides consist of the arginine, glutamine acid or aromatics rings (e.g., tryptophan, tyrosine) easily ionized. MALDI measurements have shown the presence of lactic acid (LA) dimer *m*/*z* 182.846 [2LA + H]^+^ and its metabolites: dehydrated (*m*/*z* − 18) *m*/*z* 164.854 [2LA–HOH + H]^+^ and the decarboxylate (*m*/*z* − 44) *m*/*z* 138.850 [2LA–COO + H]^+^ dimer of LA.

### Antimicrobial activity assay

**Fig. 6:**
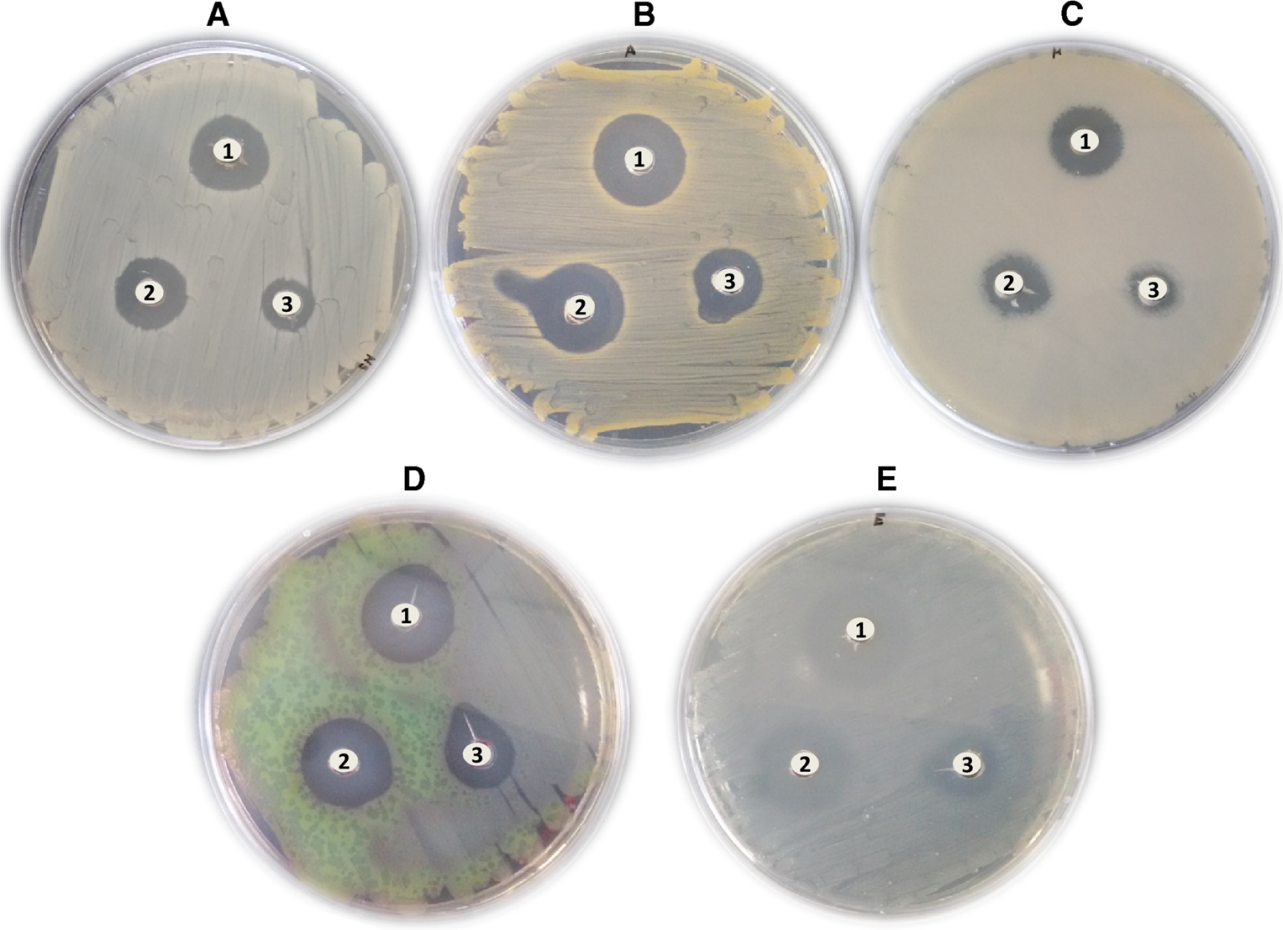
The inhibition zones of LCLB56-AgNPs provided by well diffusion method against **a** *MSSA*, **b** *Staphylococcus aureus*, **c** *Proteus mirabilis*, **d** *Pseudomonas aeruginosa*, **e** *Staphylococcus epidermidis*

**Fig. 7:**
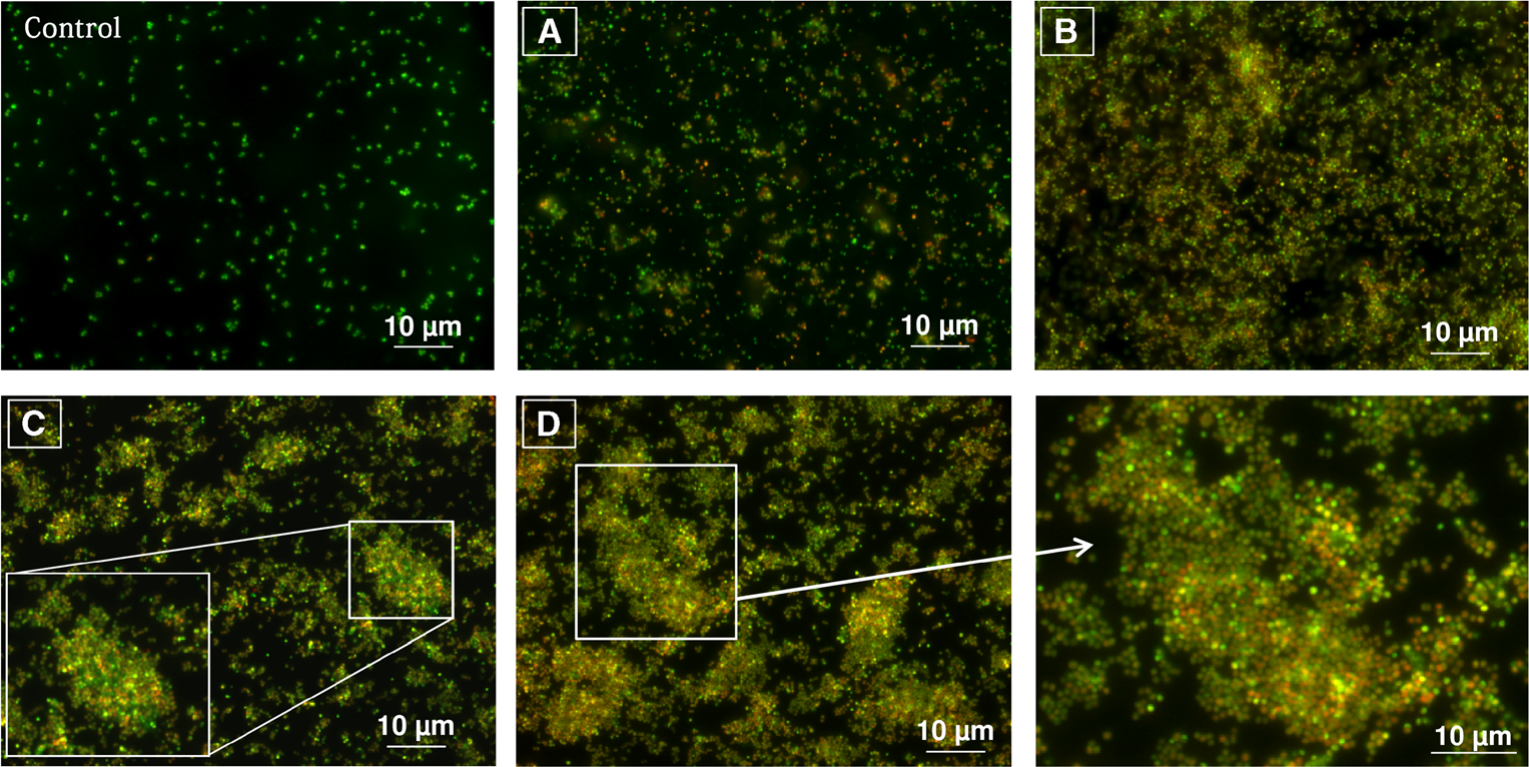
Fluorescence microscopy detection of living (*green-labeled*) and dead (*red-labeled*) *S. aureus* cells after 3 h (**a**) and 24 h (**b**) of treatment with LCLB56-AgNPs 12.5 μg/mL and after 3 h (**c**) and 24 h (**d**) with 100 μg/mL (color figure online)

LCLB56-AgNCs exerted an inhibitory effect against all tested bacterial strains (*P. aeruginosa* ATCC10145, *P. mirabilis* ATCC25933, *S. epidermidis* ATCC49461, *MSSA* ATCC29213 and *S. aureus* ATCC6338)*.* The effectiveness (inhibition zones) of LCLB56-AgCs provided by well diffusion method is shown in Fig. 6. More pronounced antimicrobial effect was noticed for 15 μg/well in comparison with 7.5 and 1.875 μg/well (Table 1). As shown in Table 2, MIC_90_ of synthesized LCLB56-AgCs was in a range of 3.125–12.5 μg/mL*. S. aureus*, *S. epidermidis*, and *P. mirabilis* were especially susceptible to LCLB56-AgCs treatment at the lowest concentration 3.125 μg/mL. For *P. aeruginosa*, concentration of 6.25 μg/mL was enough to completely inhibit its growth. The MIC_90_ toward MSSA strain was found at 12.5 μg/mL concentration. Less inhibitory effect was observed against *S. aureus* in case of highest concentration of LCLB56-AgCs (200 μg/mL) with the viability of cells about 70%. Similarly in case of 100 μg/mL concentration, cell viability was reduced to 40%. Moreover, those results are in agreement with results from fluorescence microscopy assay when the amount of total cells increase considerably compared to control after 3 and 24 h of incubation (Fig. 7). Furthermore, small agglomeration was observed for the cells treated with 100 μg/mL. However, the number of dead cells (red) increased considerably for incubation lasting 24 h. The high concentrations of BioAgCs are more susceptible to aggregation in some cases, thus triggering decreases in antimicrobial properties.

**Table 1:** Antibacterial activities of different concentrations LCLB56

Bacterial cells	Mean zone of inhibition [mm]		
	15 μg/mL (1)	7.5 μg/mL (2)	1.875 μg/mL (3)
*Pseudomonas aeruginosa*	14 ± 0.12	12 ± 0.06	7 ± 0.05
*MSSA*	12 ± 0.02	9 ± 0.05	6 ± 0.05
*Staphylococcus aureus*	14 ± 0.02	12 ± 0.09	7 ± 0.06
*Staphylococcus epidermidis*	16 ± 0.05	12 ± 0.08	8 ± 0.04
*Proteus mirabilis*	11 ± 0.07	9 ± 0.09	6 ± 0.02

**Table 2:** Minimum inhibitory concentration of the LCLB56-AgCs against various bacterial cells

Concentration (μg/mL)	*P. aeruginosa*	*S. aureus*	*MSSA*	*S. epidermidis*	*P. mirabilis*
	Inhibitory effect by cells density				
200	4.68 ± 0.02	70.21 ± 0.03	1.64 ± 0.02	4.46 ± 0.01	9.67 ± 0.02
100	2.23 ± 0.01	40.42 ± 0.02	3.21 ± 0.01	4.59 ± 0.02	5.84 ± 0.01
50	2.57 ± 0.02	1.29 ± 0.01	2.60 ± 0.01	5.30 ± 0.02	6.27 ± 0.02
25	2.90 ± 0.01	0.60 ± 0.03	1.57 ± 0.02	4.85 ± 0.03	5.95 ± 0.01
12.5	1.84 ± 0.01	2.54 ± 0.01	2.10 ± 0.01	4.20 ± 0.01	5.63 ± 0.02
6.25	2.96 ± 0.01	3.86 ± 0.01	33.46 ± 0.03	3.75 ± 0.01	5.21 ± 0.03
3.125	17.23 ± 0.03	5.90 ± 0.01	55.70 ± 0.03	4.20 ± 0.02	5.31 ± 0.02
MIC_90_	6.25	3.13	12.5	3.13	3.13

The data is presented as the mean of three replicates

#### Cytotoxicity study

**Fig. 8:**
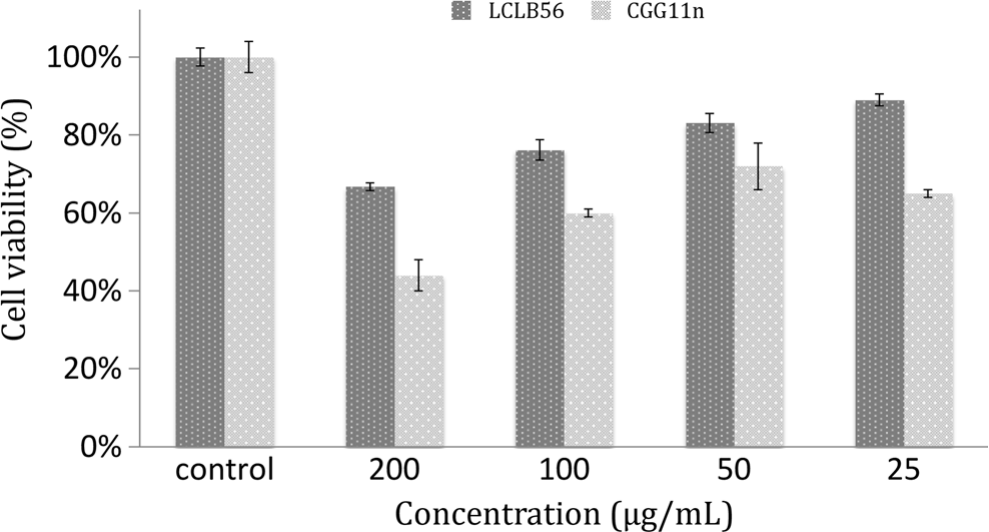
Cytotoxicity effect of silver nanocomposites synthesized by *Lactococcus lactis 56*

The in vitro cytotoxic activity of LCLB56-AgCs against mouse fibroblast cell line L929 was measured by using MTT assay. After 24 h of incubation with LCLB56-AgCs changes in cell viability were not noticed (data not shown). Only 48 h incubation leads to a reduction in cell viability. The silver composites synthesized by *L. lactis* were able to reduce viability of the L929 in a dose-dependent manner as shown in Fig. 8. For comparison, we performed the same experiment for silver biocolloids synthesized by *Actinomycete* CGG 11n (Railean-Plugaru et al. 2016). The highest used concentration of LCLB56-AgCs (200 μg/mL) reduced cell viability to 67%, whereas CGG11n to 44%. At 25 μg/mL concentration, the viability of cells was 89 and 65%, respectively. The concentration at which the cells viability was reduced to 50% for L929 cells was above 200 μg/mL for LCLB56-AgCs and 157 μg/mL for CGG 11n. However, it is worth noting that in the case of CGG11n the reduction of cell viability is not linear and lower concentration (25 μg/mL) exhibited slightly higher cytotoxicity than higher concentration (50 μg/mL).

## Discussions

Silver as a disinfectant has been known for centuries. Current environmental problems encourage researchers to search an inexpensive and more accessible safe source for synthesis of silver nanoparticles. *L. lactis* 56 is an alternative for this scope. Lactic acid bacteria are microorganisms isolated from different biological matrices, including raw milk, dairy products and fermented foods. In particular, samples of human and animal milk are habitats rich in nutrients and they are often used for isolation of a diverse spectrum of LAB (Martín et al. 2007; Carminati et al. 2014; Nejati et al. 2016). In our study, the identification of *L. lactis* was carried out by two different techniques, namely, 16S rRNA gene sequencing and intact cell MALDI–TOF MS. The first technique is commonly used for identification of LAB (Delgado et al. 2013; Neubeck et al. 2015). Recently, a growing interest of researchers in methods utilizing MALDI–TOF MS can be observed, because of its rapidity, repeatability, and precision. Currently, MALDI–TOF MS profiling of whole bacterial cells is being used more frequently for bacterial identification (Dec et al. 2014). Furthermore, based on shape of isotopic pattern of d-electrons metals, it is possible to identify the organic compounds connected with these metals and clusters (Nicolardi et al. 2010). In this work, there were observed the signals of silver isotopes [^107^Ag]^+^ *m*/*z* 106.905 and [^109^Ag]^+^ *m*/*z* 108.910. The presence of silver ions in sample can result from laser induction of ionization processes in ion source. Many of released in ion source silver ions interact both with matrix and organics part of biocolloids. The signals *m*/*z* 216.078 and 485.154 (green stripe) originate from interaction of released silver ions with matrix [HCCA + Ag]^+^ and its dimer [2HCCA + Ag]^+^ that are present in both sample and control as well. Moreover, there were registered the clusters of silver [Ag_*x*_ + Organics + H]^+^ combined with organic compounds (absent in control sample): *m*/*z* 322.720, 354.673, 2086.124, 2579.282, or 3285.183. The presence of this signal suggests that, under laser treatment in ions source, the biocolloid system is destroyed to smaller pieces of silver cluster. Moreover, the obtained results confirm the presence of lactic acid, their metabolites and organic deposit connected with silver core of nanoparticles. In opposition to other previous unpublished results realized by our group, the presence of lactic acid and its metabolites in organic of LCLB56 suggests on higher saturation area of silver core and increases the capacity of binding secondary released silver ions.

The results obtained by MALDI technique are correlated with those from FTIR and fluorescence spectroscopy assay. The spectroscopic results proved the presence of active functional groups of organics part of silver biocolloids. The emission band obtained at 493 nm under excitation at 480 nm is the effect of light interaction with silver core of nanocomposites (Kun et al. 2015; Bharat et al. 2016; Jalaluddin et al. 2016). The fluorescence of silver nanocomposites has been proposed to explain fluorescence resonance energy transfer (FRET) process and electron transfer process by several mechanisms including Purcell effects,(Kun et al. 2015; Bharat et al. 2016). Due to nanometer sizes, the silver biocolloids have a more discrete energy level as that of molecules, thus metal nanocomposites can still interact with incident light via electronic transitions between discrete energy levels, leading to strong light absorption and emission (Bharat et al. 2016; Jalaluddin et al. 2016). Furthermore, heterogeneity of silver biocolloids surface is extremely important for the optical properties of nanosystem. The interaction of surfaced ligands with silver core influence on charge–transfer process, dispersion stability, and high concentration of metal biocolloids, which results in quenching of florescence (Jalaluddin et al. 2016, Pomastowski et al. 2016). The emission band obtained at 320 nm under excitation at 280 nm is the effect of organics ligand present onto silver nanocomposites surface. Amino acids with aromatic side chains such as tyrosine, tryptophan, and phenylalanine under excitation of wavelengths in 280–295 nm are fluorescent with high quantum yields (Innocent et al. 2009; Ammor et al. 2004; Jia et al. 2015).

Another proof of present organics part in silver biocolloids system was observed during mineralization—sample preparation stage for AAS determination of silver concentration in LCLB56. Obtained dark color of silver composites, under treatment of concentrated nitric(V) acid, was changed to white-yellow precipitate. Moreover, it was also observed the drastic increase of sample volume. This phenomena could be explained by well-known xanthoproteic reaction of protein, peptides—present in organics deposit of LCLB56 system (Chatterjea 2004). Furthermore, the formation of silver nanoparticles was confirmed by SEM and TEM with high productivity of the biosynthesis. The average amount of nanoparticles was 0.363 ± 0.09 mg from 50 mL of culture medium. The synthesis efficiency of synthesis varied from 71 to 85%. The masses of dried nanoparticles vary from 1.23 to 0.95 mg from 50 mL of culture medium. It indicates that 30–37% of biocolloids constitute the silver core and 70–63% organics part.

Many studies proved the application of LAB-silver active as a strong antibacterial agent against pathogens (Shrivastava et al. 2007). This research showed effectiveness of LCLB56-AgCs against all tested Gram-positive (*S. epidermidis* ATCC49461, *MSSA* ATCC29213 and *S. aureus* ATCC6338) and Gram-negative (*P. aeruginosa* ATCC10145, *P. mirabilis* ATCC25933) bacteria. Moreover, the minimum inhibitory concentration (MIC) of LCLB 56 AgCs was relatively low against *S. aureus* (3.12 μg/mL), *P. mirabilis* (3.12 μg/mL) and *P. aeruginosa* (6.25 μg/mL) bacteria as compared to HGG16n_BioAgNPs and CGG 11n_BioAgNPs synthesized by *Actinobacteria* reported by our group in other research (Railean-Plugaru et al. 2016; Buszewski et al. 2016). Inhibitory effect of silver composites depends on the isolated strain. It seems that LCLB 56 AgCs synthesized by *L. lactis* 56 is even more effective than silver biocolloids obtained by *Actinobacteria* strain. On the another hand, the effect of silver composites could results from higher dispersion of this solution. In this regard, high dispersion seems to create colloidal stability of the system, thus increasing the bioavailability of silver. Furthermore, the presence of different organic deposit connected with silver core of nanoparticles could influence the colloid stability, consequently effectiveness of bioactive silver composites.

Many studies indicate that bioactive silver possess adverse effects on alive eukaryotic cells. It is believed that silver particles could be toxic at cellular, subcellular, and biomolecular level (Dubey et al. 2015). Hence, the first step in application of AgNPs as antibacterial agent should be evaluation of the cytotoxicity. The results indicate that cytotoxicity of silver composites synthesized by *L. lactis* 56 depends on time and concentration. After 1 day of incubation, there were no signs of cytotoxicity. The value of IC 50 after 48 exposition was above 200 μg/mL and is much higher than for example value obtained for silver nanoparticles synthesized by *Actinobacteria* CGG 11n and *Nocardiopsis valliformis* strain OT1 (Railean-Plugaru et al. 2016; Rathod et al. 2016). These results suggest that the synthesized LCLB56-AgNCs show the interest for future as a potentially safe agent for treatment of oral and external bacterial infections.
